# Returning individual research results in international direct-to-participant genomic research: results from a 31-country study

**DOI:** 10.1038/s41431-022-01103-z

**Published:** 2022-04-28

**Authors:** Michael Lang, Ma’n H. Zawati

**Affiliations:** Faculty of Medicine and Health Sciences, Centre of Genomics and Policy, McGill University, Montreal, QC Canada

**Keywords:** Genetics research, Translational research

## Abstract

This paper summarizes the results of a 31-country qualitative study of expert perspectives on the regulation of international “direct-to-participant” (DTP) genomic research. We outline how the practice of directly recruiting participants for genomic studies online complicates ethics and regulatory considerations for the return of individual research results. As part of a larger project supported by the National Human Genome Research Institute, National Institutes of Health, we prepared and distributed to 31 global legal experts a questionnaire intended to ascertain opinions and perspectives on the way international DTP genomic research is likely to be regulated. We found significant disagreement across jurisdictions on the most favorable approach to managing such results, with some countries favoring return by default and others preferring to return only with the express consent of research participants. We conclude by outlining policy considerations that should guide researcher practices in this context. As international DTP genomic research evolves, jurists and ethicists should be attentive to the ways novel approaches to subject recruitment align with existing ethical and regulatory norms in research with human participants. This paper is a preliminary step toward documenting such alignment in the context of the return of individual research results.

## Introduction

Strategies for recruiting human participants to take part in genomic research are undergoing a paradigm shift. While genomic science depends on the ability of researchers to recruit large numbers of participants to donate biospecimens and medical data [1], traditional recruitment methods are often operationally challenging, inefficient, and labor intensive [2]. Conventional studies take place in a small number of research centers and potential participants are individually identified and enrolled, usually through treating physicians, hospitals, or biobanks. This kind of approach has produced a genomic research model that is geographically restricted and that inadequately reflects the genetic diversity of the human population [3]. Research on rare disease is especially hampered by this traditional model. Low case incidences and geographic dispersion across an affected population severely limits the capacity of researchers to recruit participants and collect genetic samples [4].

One way to address these challenges might be to recruit participants directly over the Internet [5]. Online communities and consumer smartphone applications could provide access to a large pool of potential research participants. Internet-facilitated research recruitment further permits researchers to engage directly with individuals and communities who are already strongly motivated to contribute to the advancement of genomic science. Online recruitment empowers researchers to virtually consent, enroll, assess, and recontact individual participants. Kits for collecting saliva or blood samples, for example, could be shipped directly to enrolled participants and returned by post, without any requirement that participants visit a collection site in person [6]. Approaching genomic research in this way might enable participant-driven studies and citizen science to an unprecedented degree [6]. This emerging “direct-to-participant” (DTP) model of genomic research recruitment promises to vastly increase the capacity of researchers to efficiently recruit and enroll large numbers of demographically representative participants. In principle, DTP genomic research need not operate solely within the borders of a researcher’s home country. As more of the global population connects to the internet, there is a growing interest among researchers to expand genomic studies abroad [6]. While doing so would likely produce more diverse participant cohorts capable of generating important scientific discoveries, international DTP genomic research also encounters significant regulatory hurdles. It is often unclear, for example, whether researchers in one country would be permitted in law to recruit research participants in another [6]. One especially pernicious challenge in this context might be the return of research results to study participants.

Questions about whether and how to return research results, including individual and aggregate findings, are ethically and practically complex [7]. Though a vast literature has developed on the legal and ethical grounds for the return of research results [8], there remains substantial regulatory disagreement across jurisdictions over how these results ought to be managed [9]. International DTP genomic research might give this regulatory disagreement an especially challenging patina. Online systems that mediate genomic research could in some cases make the return of results more practically feasible than in conventional research. Individual data could be electronically shared, for example, by push notifications processed on a mobile application. But international DTP genomic research could also significantly complicate the return of results. Some jurisdictions might prohibit returning clinically unverified results or require deidentification that would preclude certain kinds of sharing. Researchers interested in sharing results with participants in another country may encounter uncertainty about the legality of their doing so. Participants might also be wary of receiving results generated in countries other than their own, especially if they are preliminary, unconfirmed, or ambiguous. This paper addresses these concerns by describing the findings of a 31-country qualitative study on the regulation of international DTP research with respect to the return of individual results.

## Materials and methods

We sought to understand how international DTP genomic research might affect our conventional ways of thinking about the return of research results to participants. In early 2019, we conducted a qualitative survey of legal experts in 31 countries to determine how international DTP genomic research is regulated around the world. This work was supported by the National Human Genome Research Institute, National Institutes of Health, through Grant No. 5R01HG009914-02. Survey development began in a series of three meetings attended by project co-investigators and experts in international and genomics research. Team members individually identified candidate questions to include in the survey questionnaire. Three further meetings with researchers, patient advocates, and experts on international research regulation were convened to identify thematic priorities and revise candidate survey questions. Following this, we finalized a 10–item questionnaire. The survey questions, authors, and participating countries are reported as appendices to this paper. In February 2019, we distributed the questionnaire to leading experts in research ethics and law in 31 countries. We selected experts with whom our research team had collaborated in the past, representing countries with a wide diversity of geography, population size, legal tradition, and degree of biomedical research development. We asked each of the experts to prepare a 2000-word report outlining their responses to the survey questionnaire. Respondents from Canada and the United States, countries representing the project’s principal investigators, were permitted to submit longer reports. Between March and August 2019, we iteratively reviewed the reports for completeness, comprehensibility, and form. In July 2019, the research team met to compile findings and develop recommendations. Our principal findings and completed expert reports were published in the Journal of Law, Medicine & Ethics in the winter of 2019 [10, 11]. While the findings reflect the views of leading experts in the regulation of genomic science, others in the surveyed countries might have reached different conclusions.

One item included on our initial survey (Question 8) asked about the return of research results to participants. We asked: “Does your country have laws, policies, guidelines, or cultural expectations regarding the return of individual or aggregate research results?” [11]. We provided respondents four possible responses and asked for further elaboration as appropriate. Most respondents supplemented the responses below with additional context and discussion.

- The law requires the return of individual results unless the participant expressly declines to have results returned.
- The law is silent on return of results; the expectation is that individual results will be returned unless the participant expressly declines to have the results returned.
- The law is silent on return of results; aggregate results are typically returned, but individual results are not returned unless expressly stated in the research protocol.
- I am not sure — or other answer.

Our initial 2019 report did not detail findings with respect to this question, nor did we pose recommendations specifically intended to facilitate the management of the return of individual results. In late 2020, we revisited our initial dataset to determine whether our findings could serve to clarify how the return of results to participants is regulated in the 31 target countries and how the conduct of international DTP genomic research is likely to complicate existing practices. After an initial assessment of the existing dataset, we developed the following research question: How does the law structure the return of individual findings in the surveyed countries and how do the applicable standards affect the feasibility of conducting international DTP genomic research? We then reviewed the expert report dataset, focussing primarily on Question 8, and tabulated the results below.

## Results

We found disagreement among respondents about the likely regulation of the return of research results in the context of international DTP genomic research. Nine experts indicated that the law in their country requires the return of individual results unless a participant expressly declines to receive them. Five experts indicated that the law is silent but that participants generally expect to have individual results returned unless they explicitly decline. Five others indicated that the law is silent but there is an expectation that individual results should not be returned. Twelve reported another response. The figure below summarizes these findings. More detailed expert responses are provided in Appendix 2.

Respondents sketched out a complex and highly varied regulatory picture. In some jurisdictions, researchers are only required to indicate whether results will or will not be returned during the consent process. In other countries, aggregate research results are not typically returned whereas significant individual results are. In some countries, law, policy, guidance, or culture generate a presumption that results are to be returned. In other jurisdictions, the prevailing presumption operated with a contrary effect. Even within the individual response categories above, we found a high degree of variance in the experts’ written responses. Nine experts reported that the law in their jurisdiction requires the return of individual results unless the participant declines explicitly. The countries taking this position were Australia, Brazil, Denmark, India, Peru, Qatar, South Africa, Spain, and Switzerland. While Australian researchers are “required to consider” [10] whether to return research results, for example, Brazil’s Guidelines for Ethical Analysis of Human Genetic Research Projects requires the universal return of individual results unless a participant has explicitly declined to receive them [10]. In Peru, regulations specify that both individual and aggregate results must be returned. Other provisions underline the importance of community benefit sharing [10]. Swiss law similarly provides a right “to be informed of results relating [to health]” and specifies that the communication of results must be carried out in a manner appropriate to the circumstance [10]. In Denmark, rules on the return of results are governed by an Executive order of the Ministry of Health, which specifies that researchers must inform participants if important information about their health is discovered. There are only narrow exceptions to this rule, such as when a participant has clearly indicated that they do not wish to receive such information [10]. Researchers in Denmark are also mandated to return general research results along with a summary of potential implications for individual participants, so long as it is practically feasible to do so.

Five further countries report that the law is silent on the return of individual research results, but that there is an expectation that individual results will be returned unless the participant has declined to receive them. Countries taking this approach are Canada, China, Mexico, Nigeria, and Uganda. In the Chinese context, for example, no law, policy, or guidance regulates the return of results. But research participants and research ethics boards generally expect that results will be returned unless participants decline to receive them [10]. This is the same basic approach taken in Mexico, with the additional specification that Mexican law does not comment on the return of results for international research [10].

Five jurisdictions have no explicit treatment of the return of individual results in domestic law but typically return aggregate findings: Greece, Israel, Italy, Jordan, and Poland. In Israel, researchers will often return aggregate results. Individual results are typically only returned if this is explicitly contemplated in the research protocol, though the national research ethics board does expect that researchers are capable of returning actionable research results [10]. Italy takes a similar approach. Its Committee for Bioethics underscores that the return of individualized information in the context of large scale research projects will usually be infeasible. The Committee further specifies an expectation that clinically relevant results will be returned if requested by the research participant [10]. Jordan’s expert notes that there is broad interest in the population in the return of individual results with a preference for an opt–in regime integrated into the informed consent process [10].

Nearly 40%, a strong plurality of experts, did not select one of the responses above. Twelve countries expressed a more nuanced position or reported uncertainty about regulatory requirements for the return of results. Experts taking this approach were Estonia, Finland, France, Germany, Japan, the Netherlands, Singapore, South Korea, Sweden, Taiwan, the United Kingdom, and the United States. Of these, Sweden and the United Kingdom did not provide additional comments, potentially indicating that the expectations and rules surrounding the return of results are uncertain [10]. Likely the most complex regime of any surveyed country is that of the United States, in which there is an active and ongoing debate about “the extent to which individual research results should be returned to participants” [10].

Three principal mechanisms provide for the return of individual results in the United States: (a) research analysis in a laboratory compliant with the Clinical Laboratory Improvement Amendments (CLIA), which permits research findings to be freely used in clinical care, (b) results confirmation in a CLIA–compliant lab, or (c) clinical handoff, in which research results are returned on the advice that a CLIA–compliant lab confirm their validity before they are used to inform clinical care [10]. CLIA’s most significant impact on the return of results is its prohibition on providing results for the diagnosis, treatment, or management of disease by uncertified laboratories [10].

Probably the clearest enunciation of a position on the return of results in US federal law or policy is found in the Revised Common Rule, which requires that researchers specify whether results will be returned as an element of the informed consent process [10]. Beyond federal law, several organizational guidance documents provide a certain degree of clarity on the return of results in the United States. The American College of Medical Genetics and Genomics (ACMG), for example, maintains a list of gene variants that should screened for and returned to research participants with their consent whenever any genome sequencing is undertaken [10]. These guidelines apply only to the communication of clinical results. Certain scholars have nevertheless suggested that the guidelines could be adopted in the research context as well [10].

There is similar debate over what the law requires in Finland and Japan. Finland’s rules do not specify whether provisions allowing participants to request research results applies to raw genomic data [10]. Researchers in Japan generally do not return research findings, but ethical guidelines for research in the genetics context permits participants to request individual results. A researcher not wanting to engage in return must clearly communicate their reasons for this position [10]. Other jurisdictions take a more direct approach. Singapore, for example, requires that researchers specify as part of the informed consent process whether and to what extent results may be returned [10]. South Korea’s rules work in a similar way, though there is a widespread expectation among the public that, in practice, results will generally be returned [10]. In Germany, the law privileges informational determination, implying a right to know results that constitute personal data, as well as a corollary right not to know [10]. The practice in Germany is to generally return results unless a participant has declined to receive them. This closely follows the approach taken in Estonia, in which there is a strong cultural expectation that results will be returned. Research participants have both a right to know and right not to know if they choose [10]. France, likewise, requires the return of individual results unless a participant explicitly declines [10].

## Discussion

In our prior publication, we detailed significant disagreement among the surveyed experts on regulatory mechanisms likely to apply to international DTP genomic research [11]. In part, this is likely owing to the relative novelty of international DTP genomic research and the absence of specific regulation or guidance controlling the field. We found, for example, that none of the surveyed countries have legal or regulatory instruments in place that specifically contemplate DTP genomic research and that the likely application of existing rules surrounding research ethics requirements in this space were highly variable. Seventeen experts indicated that a foreign researcher wanting to recruit participants in their country would require local ethics approval, even if the study had been approved by a research ethics board in the researcher’s home jurisdiction [11]. Five experts reported that foreign researchers would be permitted to conduct DTP genomic research with ethics approval in the researcher’s home jurisdiction even without local ethics approval. Nine others were unsure. With this conflicting backdrop in mind, our team recommended that international DTP research be subject to a regime of single site ethics review [11]. This recommendation follows from the “fundamental agreement of ethics policies around the world” and is intended to help promote greater efficiency and consistency research ethics decisions applicable to international DTP genomic research [11]. We argued that (1) where an international DTP genomic research intervention is approved by an ethics review body in the researcher’s country and (2) an ethics review body in the participant’s country has determined that ethics review policies in the researcher’s jurisdiction are adequate, then (3) ethics approval in the researcher’s country should be considered valid in the participant’s country [11]. We also noted that additional research is required to clarify the impact of divergent cultural and religious practices on the future regulation of international DTP genomic research.

Debate surrounding the ethics and regulation of the return of individual research results has flourished in recent years [12]. Commenters vary considerably in their assessment of whether and under what conditions results should be communicated to participants. Though participants often report a strong interest in accessing results derived from their participation in research [13], a variety of ethical arguments both support and oppose permitting such access. Questions about the return of research results in the context of international DTP genomic research are especially complex. Researchers implementing international online recruitment strategies will need to account for wildly divergent rules, practices, and expectations. In our previous work on international DTP research, we proposed a system of single site ethics review. Our findings here suggest that this approach may be applied with a high degree of flexibility when considering the return of research results. As we set out above, jurisdictions typically take one of three possible approaches to the return of individual research results: requirement, prohibition, or discretion. Most surveyed experts report that the return of results is discretionary. In these countries, attitudes differ about whether individual results should be returned as a default position, but the law does not generally offer a specific prescription.

In ten of the jurisdictions we surveyed, including Canada, Mexico, Italy, and Jordan, the law is formally silent on these questions, but researchers will often return individual results that meet a certain set of criteria, such as clinical validity or actionability, depending on participant preferences. In nine of the countries we surveyed, including India and Switzerland, researchers are legally required to return individual results, though participants are usually able to expressly decline to receive such results, as in the case of Denmark. None of the countries we surveyed unambiguously prohibit the return of individual results. Certain US scholars argue that returning individual research results might be prohibited in one specific instance: when results are obtained from a non-CLIA certified laboratory and are returned for the use in the diagnosis or treatment of disease [10]. This is as close to an outright prohibition on the return of results that we found in this study, though it is certainly conceivable that jurisdictions not surveyed might take a stricter approach. Importantly, though, this perspective is not universally held. Other scholars suggest that CLIA has no application where results are returned for the purpose of prompting a participant to seek follow-up care and clinical confirmation of research findings [10]. Similar disagreement surrounds the Health Insurance Portability and Accountability Act (HIPAA), which may be interpreted to provide a right of access to individual research results [10].

This diversity of approaches likely generates uncertainty among researchers and research ethics boards reviewing international DTP genomic research protocols, which in turn might have the effect of stifling research activity, producing contradictory practices, or encouraging the inappropriate communication or withholding of results. Of course, other factors are likely to significantly affect practices surrounding return. For one thing, the practicability of returning results will depend in large measure on a researcher’s approach to data management. If collected samples and data are anonymized or de-identified, for example, returning individual research results might not be possible. Considering that data processing and results communications norms differ markedly across borders, knowing whether and how to facilitate return may prove to be a highly multifaceted problem. For another thing, international DTP genomic research might be especially prone to the production of results that are uncertain or ambiguous. These projects, after all, will often assess large, highly heterogenous populations from which it may be technically challenging to draw out clear, individually targeted findings. International DTP genomic research will also likely facilitate participant and citizen directed research, some of which may not be principally concerned with producing findings that are clearly determinative at the level of the individual. Researchers may also be unaware of the cultural or social significance of the return of results in a country in which they have enrolled participants. Finally, obligations and expectations with respect to the return of results will depend significantly on the nature of the results in question, for example, whether the results are medically actionable or whether the research participant is a minor at the time of enrollment. What researchers are required to do in contemplation of these kinds of results may be uncertain across jurisdictions: Table 1.

**Table 1:** Responses to survey Question 8.

Rules and expectations for the return of research results	
a. The law requires the return of individual results unless the participant expressly declines to have results returned.	9 (29%)
b. The law is silent on return of results; the expectation is that individual results will be returned unless the participant expressly declines to have the results returned.	5 (16%)
c. The law is silent on return of results; aggregate results are typically returned, but individual results are not returned unless expressly stated in the research protocol.	5 (16%)
d. I am not sure — or other answer.	12 (39%)

These conditions will sometimes combine to make the generalized return of research results in the international DTP genomic research context unfavorable. In the hypothetical case that a country prohibits the return of individual research results, for example, foreign researchers will be compelled by regulation not to engage in their ususal practices. While the regime of single site ethics review that we defend elsewhere would help to facilitate international DTP genomic research, our findings here suggest that a system of this kind should be supplemented with specific guidance for the management of aggregate and individual research findings. We propose thinking about the management of the return of individual research results according to common regulatory elements across the jurisdictions in which international DTP genomic research occurs. Unsurprisingly, the easiest cases are those with jurisdictional agreement. Substantively similar regulation would tend to produce straightforward researcher obligations. An international DTP genomic study conducted in jurisdictions that all require return, for example, might differ in only marginally in respect of when or under what conditions individual results are communicated. Likewise, jurisdictions in which return is discretionary are likely to align on most features of their return regimes. We found, for example, that these kinds of countries usually require that researchers clearly inform participants whether individual results will be shared. Participants might alternatively be given an opportunity to expressly decline or opt-into the receipt of individual results. In deciding whether to return individual results in jurisdictions that make such return discretionary, investigators ought to consider the degree to which the communication of individual results, owing to funding or organizational constraints for example, is practicable. It may not always be possible, depending on a study’s ambit or resources, to systematically return individual research results to all participants. Researchers should generally work to ensure that any legal requirement or optional commitment to return results can feasibly be discharged over the duration of the project. Researchers might also consider informing participants that returned results may need clinical validation before they are used in care decisions. In effect, and despite these limits, international DTP genomic research that occurs in two or more jurisdictions taking an identical regulatory approach to the return of individual results may simply adopt the practices dominant in the researcher’s home country.

It is significantly more challenging to understand how the return of results should be addressed when regulation differs in two or more jurisdictions. Researchers operating in countries that take conflicting approaches should be attentive to the potential disparity in returning individual results to participants in one country while declining to do so for participants in another, which would have the effect of treating research participants differently according to a contingent factor: their country of residence. As a general proposition, we propose that researchers should adopt, where possible, an approach capable of accommodating regulation in each location. This will often require implementing something that resembles the most restrictive available approach to return. Suppose, for example, that a study recruits from two countries: one in which return is required by law and another in which it is discretionary. Clear consent language may be designed such that participants in both jurisdictions are provided an option to expressly decline to receive individual results. Alternatively, participants in the country with discretionary return may consent to receive results, and language to this effect may be included in the research protocol. In either case, the informed consent process will serve as a useful tool for structuring participant expectations and ensuring that researchers comply with applicable law and local practice. A related set of challenges might exist when return is organizationally impractical in one or more of the countries implicated in international DTP research but is not prohibited in any. These otherwise intractable scenarios could benefit from coordination between researchers and research ethics bodies. Local research ethics authorities are, after all, typically well placed to ensure research fairness and protection of the interests of participants. This kind of coordination could result in the practical accommodation of otherwise divergent regulations and practices. Coordination could be particularly helpful in the most challenging set of scenarios predicted by our findings, when research is conducted in two jurisdictions: one that requires return and one that prohibits it. On the surface, these positions might appear irreconcilable. But research ethics boards may be well placed to navigate the applicable rules and, where necessary, carve out the right exceptions.

Specific variation in the regulation of return of results will naturally require careful attention. In practice, the system of single site ethics review we previously proposed may suggest a solution to coordinating the return of results. In our 2019 report, we noted that a regime of single site ethics review could become legally binding though unilateral recognition. According to this model, an international DTP project could be deemed to comply with local law and custom if the project has been approved by the relevant ethics authority in the researcher’s country and the participant’s country has determined that ethics review in the researcher’s country is adequately compliant with local law, policy, and practice. The United States, for example, could determine that ethics review in Canada is equivalent to ethics review in the United States for the purposes of the evaluation of an international DTP genomic research project and thus adequate to satisfy requirements under the Common Rule [11]. Such determinations, made in full contemplation of a reciprocal jurisdiction’s approach to the return of research results, could potentially accommodate divergent rules by being expressly dependent, among other things, on a particular approach to the return of results. In any case, a single global approach to managing the return of results in the context of international DTP genomic research is likely at present to be unworkable. It is, we think, preferable that countries in which there is significant interest in international DTP genomic research set mutual standards as between themselves and peer countries through the system of unilaterally approved single site ethics review that we have elsewhere defended. Until rules and practices surrounding the return of results move toward some degree of international clarity and harmonization, this kind of approach may be an efficient way to facilitate international DTP genomic research that affirms both participant autonomy and the internationally divergent regulatory infrastructure that modulates the return of results. For researchers conducting international DTP genomic research in the absence of mutual ethics recognition, it is likely prudent, and in many cases legally required, for researchers to ensure compliance with law, policy, and practice in jurisdictions from which participants are recruited. What this means in practice is that researchers may require local collaborators in participant jurisdictions, as well as local ethics approval prior to recruitment. This approach will naturally be administratively burdensome, underscoring the necessity of policy that responds proactively to these emerging trends.

## Conclusions

This paper summarizes the regulation of the return of research results for international DTP research in 31 countries around the world. The surveyed jurisdictions reflect both a broad diversity of approaches and presumptions concerning whether and in what manner research results ought to be returned. This jurisdictional diversity is reflected in policy guidance recently adopted by the Global Alliance for Genomics and Health (GA4GH). GA4GH notes that whether and how clinically actionable research results are returned should be guided by local practices [14]. GA4GH further suggests that practices surrounding the return of results should be informed by precise protocol language and should be tailored to relevant participant communities [14]. GA4GH similarly advocates for ethics review reciprocity based requisite common elements of ethics review on in its 2017 policy on ethics review recognition [15]. The emerging international consensus on the conditions under which certain individual research results ought to be returned is, we think, compatible with the approach we have proposed in this paper. We suggest that a system of single site ethics review through unilateral recognition of regulatory equivalence for the approval of international DTP genomic research may accommodate diverging standards on the return of research results. As international DTP genomic research becomes increasingly popular, further research must be conducted to better understand how participant interests can be advanced and protected.

## Supplementary information

Appendix 1

Appendix 2

Appendix 3

Supplementary File

## Data Availability

Study data is included in supplementary files to this paper.

## References

[1] Navarro FCP, Mohsen H, Yan C, Li S, Gu M, Meyerson W (2019). Genomics and data science: an application within an umbrella. Genome Biol.

[2] Sharif SM (2020). Enhancing inclusion of diverse populations in genomics: A competence framework. J Genet Counseling.

[3] Ben-Eghan C, Sun R, Hleap JS, Diaz-Papkovich A, Munter HM, Grant AV (2020). Don’t ignore genetic data from minority populations. Nature.

[4] Kempf L, Goldsmith JC, Temple R (2017). Challenges of developing and conducting clinical trials in rare disorders. Am J Med Genet.

[5] Juraschek SP, Plante TB, Charleston J, Miller ER, Yeh H, Appel LJ (2018). Use of online recruitment strategies in a randomized trial of cancer survivors. Clin Trials.

[6] Rothstein MA, Zawati MH, Knoppers BM (2019). Regulatory landscape of international direct-to- participant (DTP) genomic research: time to untie the gordian knot?. J L Med Ethics.

[7] Thorogood A, Dalpé G, Knoppers BM (2019). Return of individual genomic research results: are laws and policies keeping step?. Eur J Hum Genet.

[8] Wolf SM (2020). Return of results in participant-driven research: learning from transformative research models. J L Med Ethics.

[9] Lévesque E, Joly Y, Simard J (2011). Return of research results: general principles and international perspectives. J L Med Ethics.

[10] Zawati MH (2019). Country reports. J L Med Ethics.

[11] Rothstein MA, Zawati MH, Beskow LM, Brelsford KM, Brothers KB, Hammack-Aviran CM (2019). Legal and ethical challenges of direct–to–participant genomic research: conclusions and recommendations. J L Med Ethics.

[12] Wolf SM, Evans BJ (2018). Return of results and data to study participants. Science.

[13] Illes J, Kirschen MP, Edwards W, Stanford LR, Bandettini P, Cho MK (2006). Incidental findings in brain imaging research. Science.

[14] Global Alliance for Genomics and Health, “2021 Policy on Clinically Actionable Genomic Research Results” (2021) POL 007v1.0.

[15] Global Alliance for Genomics and Health, “Ethics Review Recognition Policy” (2017) POL 004 / v. 1.0.

