# Supplementary material for: Returning individual research results in international direct-to-participant genomic research: results from a 31-country study
Source: Eur J Hum Genet. 2022 Apr 28;30(10):1132–7. doi: 10.1038/s41431-022-01103-z (PMC9553878; doi:10.1038/s41431-022-01103-z)
Supplement: Supplementary file 1 — Appendix 1 [file 41431_2022_1103_MOESM1_ESM.docx]

**Appendix 1: Participating countries**

| ***Asia*** | China, India, Israel, Japan, Jordan, Qatar, Singapore, South Korea, Taiwan |
| --- | --- |
| ***Africa*** | Nigeria, South Africa, Uganda |
| ***Europe*** | Denmark, Estonia, Finland, France, Germany, Greece, Italy, Netherlands, Poland, Spain, Sweden, Switzerland, United Kingdom |
| ***North America*** | Canada, Mexico, United States |
| ***South America*** | Brazil, Peru |
| ***Oceania*** | Australia |
