# Supplementary material for: Returning individual research results in international direct-to-participant genomic research: results from a 31-country study
Source: Eur J Hum Genet. 2022 Apr 28;30(10):1132–7. doi: 10.1038/s41431-022-01103-z (PMC9553878; doi:10.1038/s41431-022-01103-z)
Supplement: Supplementary file 2 — Appendix 2 [file 41431_2022_1103_MOESM2_ESM.docx]

**Appendix 2: Report authors**

| ***Country Reports*** | ***Authors*** |
| --- | --- |
| Australia | Don Chalmers (AO, FA AL, FAHMS). |
| Brazil | Sueli G. Dallari (MSc, PhD) & Marina de Neiva Borba (MSc, PhD). |
| Canada | Miriam Pinkesz (BCL, LLB) & Yann Joly (PhD). |
| China | Haidan Chen (PhD). |
| Denmark | Mette Hartlev (PhD, LLD). |
| Estonia | Liis Leitsalu (PhD). |
| Finland | Sirpa Soini (LLM). |
| France | Emmanuelle Rial–Sebbag (PhD). |
| Germany | Nils Hoppe (LLB, Dr. iur). |
| Greece | Tina Garani-Papadatos (PhD) & Panagitotis Vidalis (PhD). |
| India | Krishna Ravi Srinivas (PhD). |
| Israel | Gil Segal (MD, LLB, SJD). |
| Italy | Stefania Negri (PhD). |
| Japan | Ryoko Hatanaka |
| Jordan | Maysa Al-Hussaini (MD, FRCPath) & Amal Al-Tabba’ (MSc). |
| Mexico | Lourdes Motta-Murgía (MSc, LLB, PhD) & Laura Estela Torres Moran (LLB, LLM). |
| Netherlands | Aart Hendriks (PhD). |
| Nigeria | Obiajulu Nnamuchi (LLB, LLM, MA, SJD). |
| Peru | Rosario Isasi (JD, MPH). |
| Poland | Dorota Krekora-Zajac (JD). |
| Qatar | Eman Sadoun (PhD). |
| Singapore | Calvin Ho (BSc, LLB, MSc, LLM, JSD). |
| South Africa | Pamela Andanda (LLB, LLM, PhD) |
| South Korea | Won Bok Lee (MD, LLM, SJD). |
| Spain | Pilar Nicolás (PhD). |
| Sweden | Titti Mattsson (PhD). |
| Switzerland | Vladislava Talanova (MLaw), Alexandre Dosch (MLaw) & Dominique Sprumont (PhD). |
| Taiwan | Chien-Te Fan (JD, LLM) & Tzu-Hsun Hung (LLM). |
| Uganda | Obiajulu Nnamuchi (LLB, LLM, MA, SJD). |
| United Kingdom | Jane Kaye (LLB, DPhil), Andelka Phillips (BA, LLB, BA, LLM, DPhil), Heather Gowans (PhD) & Nisha Shah (BSc, MSc). |
| United States | James W. Hazel (JD, PhD). |
