# Supplementary material for: Returning individual research results in international direct-to-participant genomic research: results from a 31-country study
Source: Eur J Hum Genet. 2022 Apr 28;30(10):1132–7. doi: 10.1038/s41431-022-01103-z (PMC9553878; doi:10.1038/s41431-022-01103-z)
Supplement: Supplementary file 3 — Appendix 3 [file 41431_2022_1103_MOESM3_ESM.docx]

**Appendix 3: Survey Questionnaire**

| ***Questions*** | ***Multiple Choice Responses*** |
| --- | --- |
| 1. As far as you know, is DTP genomic research a topic of interest to researchers or other stakeholders in your country? | 1. There has been little, if any, discussion of the issue as of now 2. There has been discussion among researchers, but little discussion among policy makers 3. There has been discussion among both researchers and policy makers 4. I am not sure – or other answer |
| 1. Assume that a researcher in your country wants to conduct DTP genomic research with participants in your country and that such research is subject to IRB/REC review. Please describe the conditions for IRB/REC approval, if it could be approved at all. | [free text response permitted] |
| 1. Assume that a researcher in your country wants to conduct DTP genomic research in another country. Please describe the conditions that must be satisfied for IRB/REC approval in your country, if it could be approved at all. Would your IRB/REC also require approval from a research ethics review body in the other country? | [free text response permitted] |
| 1. Assume that a researcher from outside your country wants to conduct DTP genomic research in your country. 2. Would it be lawful for the researcher to do so without IRB/REC approval in either the researcher’s country or your country? [Yes/No] 3. Would it be lawful for the researcher to do so if the research were approved by an IRB/REC in the researcher’s own country, but was not submitted for approval in your country? [Yes/No] 4. Would the external researcher be required to have a collaborator in your country? [Yes/No] 5. Would it matter whether the external researcher is based at a commercial, governmental, or academic entity? [Yes/No] | 1. Yes 2. No 3. Other/Not sure |
| 1. As far as you know, what are the perceived benefits and risks that could occur if a researcher from another country conducted IRB/REC-approved genomic research on samples or data obtained from your country? Please consider the perspectives of the public, research participants, socially defined groups (e.g., indigenous or minority populations), researchers, and other professional or government entities. | 1. Yes 2. No 3. Other/Not sure |
| 1. Does your country have biohazard committees, data protection boards, export permit authorities, or other entities that regulate the exporting of biospecimens or the transferring of data across borders for research? If so, do these requirements apply to individual citizens as well as research and medical institutions? | [free text response permitted] |
| 1. Does your country have laws, policies, or guidelines dealing with genetic or genomic research or genetic or genomic privacy that would apply to international DTP research? Do your national laws on these issues apply outside of your country when residents or citizens of your country enroll in a DTP study conducted abroad? | [free text response permitted] |
| 1. Does your country have laws, policies, guidelines, or cultural expectations regarding the return of individual or aggregate research results? [Multiple choice] | 1. The law requires the return of individual results unless the participant expressly declines to have results returned 2. The law is silent on return of results; the expectation is that individual results will be returned unless the participant expressly declines to have the results returned 3. The law is silent on return of results; aggregate results are typically returned, but individual results are not returned unless expressly stated in the research protocol 4. I am not sure – or other answer |
| 1. Does your country have laws, policies, or guidelines regarding “direct-to-consumer" genetic testing (e.g., 23andMe) and, if so, what do they provide? | 1. Yes. Direct-to-consumer genetic testing is illegal 2. Yes. Direct-to-consumer genetic testing is legal 3. No. Direct-to-consumer genetic testing is not an issue 4. I am not sure – or other answer |
| 1. How, if at all, do you anticipate that your country’s laws, policies, or guidelines will change in the next 5-10 years in response to international DTP genomic research? [Multiple choice] | 1. I do not think they will change at all 2. I think they will restrict international DTP research 3. I think they will allow international DTP research 4. I am not sure – or other answer |
