# Supplementary material for: Returning individual research results in international direct-to-participant genomic research: results from a 31-country study
Source: Eur J Hum Genet. 2022 Apr 28;30(10):1132–7. doi: 10.1038/s41431-022-01103-z (PMC9553878; doi:10.1038/s41431-022-01103-z)
Supplement: Supplementary file 4 — Supplementary File [file 41431_2022_1103_MOESM4_ESM.docx]

**Supplementary Material: Survey responses, law and expectations for the return of results**

| ***Country*** | ***Response*** |
| --- | --- |
| Australia | a. *The law requires the return of individual results unless the participant expressly declines to have results returned.*  In Australia, the return of results is a requirement of our *National Statement* in Chapter 3.3.26 where researchers are required to *consider* whether to return results of research, and in so doing, researchers should distinguish between individual and overall research results, how these results will be provided to participants, how the return of results will be managed, and the risks of the return of individual research results and overall research results. |
| Brazil | a. *The law requires the return of individual results unless the participant expressly declines to have results returned.*  In Brazil, the Guidelines for Ethical Analysis of Human Genetic Research Projects determine in the Informed Consent Form (TCLE) that all participants will be informed of all the results of exams and tests performed, but they have the option of not knowing this information, according to item V.1 of the Resolution of the Conselho Nacional de Saúde (CNS) n. 340, dated July 8, 2004. |
| Canada | b. *The law is silent on return of results; the expectation is that individual results will be returned unless the participant expressly declines to have the results returned.*  According to the TCPS2, researchers have an obligation to disclose any material incidental finding in the course of research. Typically, researchers are expected to develop a plan for how incidental findings will be disclosed to research participants, especially in genomic and genetic research. Generally, the Canadian perspective on the matter is that results should be returned where they meet the following criteria: scientific and clinical validity, significant health implications, actionability, research results confirmed by an accredited clinical diagnostic laboratory, and independent REB approval was obtained. |
| China | b. *The law is silent on return of results; the expectation is that individual results will be returned unless the participant expressly declines to have the results returned.*  c. *The law is silent on return of results; aggregate results are typically returned, but individual results are not returned unless expressly stated in the research protocol.*  China does not have laws, policies, or guidelines regarding the return of individual or aggregate research results. Most Chinese research participants usually expect for both individual and aggregate research results. IRB/RECs in China expect that individual results will be returned unless the participant expressly declines to have the results returned. Researchers in China expect that aggregate results are typically returned, but individual results are not returned unless expressly stated in the research protocol. |
| Denmark | a. *The law requires the return of individual results unless the participant expressly declines to have results returned.*  The Ministry of Health has issued an Executive Order on Information and Consent to Participants in Health Research Projects. Section 15 of the Executive Order provides that the investigator must inform the research participant if important information about the health of the research participant is found. Only in the exceptional situations where the research participant has clearly opted out of receiving such findings is the investigator not permitted to inform the research participant. Such an opt-out is only valid if it is an informed opt-out based on current and relevant insight, cf. the standard of good information practice. In addition, section 16.3 of the Executive Order also makes it mandatory for researchers — if practically feasible — to inform research participants (if they consent) about the general results of the project and of the possible consequences for the individual participant. In regards to genetic research, the National Committee on Health Research Ethics has adopted special guidelines5 making it mandatory to return certain secondary findings to participants, who — due to the options of having an exemption from the consent requirement — have not provided an explicit consent for biobank research involving comprehensive genetic analyses. |
| Estonia | d. *I am not sure — or other answer*  The cultural expectations are in favor of receiving genetic test results. Surveys carried out repeatedly have shown that approximately 75% of the adult population is interested in genetic testing in general. According to the Estonian HGRA, population biobank participants have the right to know, as well as the right not to know what data the population biobank has collected. How- ever, besides mentioning the right to counseling, it is not specified how participants should receive results or what type of results. Return of results has currently been offered on a project basis. Additionally, the GDPR Article 20 covers the right to data portability. In the context of genetic research, it would mean that the research participants providing samples for research also have the right to obtain raw genetic data generated. |
| Finland | d. *I am not sure — or other answer*  Only the *Finnish Biobank Act* §396 contains legal rules on returning results and is applied if the sample donor asks for health-related information determined from his or her sample. There is confusion as how to interpret this paragraph and if it covers raw genomic data. In addition, the sample donors may express their wish to be informed about clinically action- able research findings in the consent process. The biobank community is currently on its own initiative piloting ways and the feasibility of returning certain well-established genomic information to sample donors. There are still open questions to tackle, such as validation of results and need for counseling, integration of health care, and costs. The new Genome Centre is expected to develop procedures and give guidance in this matter. |
| France | d. *I am not sure — or other answer*  The law requires the return of individual results unless the participant expressly declines to have results returned (PHC, Article L1122-1).  Global results can also be returned (PHC, Article L1122-1). |
| Germany | d. *I am not sure — or other answer*  The law in relation to informational self-determination11 provides that individuals have a *prima facie* entitlement to know results that constitute their personal data (i.e., individual results or incidental findings). It is generally accepted that this right to know one’s own information also includes the right *not* to know the same information. Current jurisprudence, based on constitutional principles, therefore provides that information ought to be provided unless the individual has asked not to be informed. Most informed consent procedures now contain an appropriate section where individuals can opt in or out of the return of individual results. The guidelines issued by professional bodies, for example in the context of biobanking, provide sample consent procedures in line with the jurisprudence. In terms of the cul- tural expectation, it is safe to say that this is linked to the jurisprudence: the judgment which gave rise to the right of informational self-determination concerned public disquiet about the amount of information gathered in the 1980s German census. Since then, the public perception has been strongly in favor of individuals having unfettered access to their individual information, as well as strong con- trol rights in that information. This is now increasingly reflected in the provisions of the EU-GDPR (which was decisively driven and shaped by a German Green MEP). |
| Greece | c. *The law is silent on return of results; aggregate results are typically returned, but individual results are not returned unless expressly stated in the research protocol.*  Research protocols have to make provisions in accordance with the existing framework guiding research, both with regard to research ethics rules as well as research integrity. |
| India | a. *The law requires the return of individual results unless the participant expressly declines to have results returned.* |
| Israel | c. *The law is silent on return of results; aggregate results are typically returned, but individual results are not returned unless expressly stated in the research protocol.*  Aggregate results are [not necessarily] returned, but individual results are not returned unless expressly stated in  the research protocol. The national IRB does expect, and so conditions its approval, that researchers demonstrate their ability to report back to participants/patients if actionable results emerge from the study, at the expense of the researcher. |
| Italy | c. *The law is silent on return of results; aggregate results are typically returned, but individual results are not returned unless expressly stated in the research protocol.*  In the opinion issued by the Italian Committee for Bioethics on *Managing “Incidental Findings” in Genomic Investigations with New Technology Platforms*, the issue of return of information to donors of biological samples for research purposes is partly addressed. The Commit- tee observes that, in case of research involving the collection of a large number of samples, it is unrealistic to re-contact the donors to update them on the results, which could hardly have a clinical value of individual interest. The NBC recommends, however, that it should always be specified in the informed consent form whether there is this possibility and, if so, the choice of the information that one wishes to receive is left to the interested party. The Committee states that it is morally compulsory to guarantee, if requested, a return of the results of clinical relevance to patients suffering from rare diseases still lacking a certain diagnosis, who have entered into research protocols and donated their samples in the hope of accelerating their knowledge of the causes of their illness.  In the *Guidelines* it is recommended that in drafting or assessing clinical research protocols in genetics it should be borne in mind that: individual genetic results must be disclosed to the patient who requests them regardless of their possible clinical utility; individual genetic results should not be given to others if they are not of immediate clinical utility; individual results that may be useful for the health of the subject must be provided to his doctor; the investigators participating in the research should receive a report containing the global results. |
| Japan | d. *I am not sure — or other answer*  In principle, the results of genome research are not generally returned.  However, in February 2013, the “Ethical Guidelines on Human Genome and Genetic Analysis Research” were revised, and provide that, if a provider of genetic information wishes to have their research results returned, the researcher should do so. If the researcher opts not to return the results, the reasons or conditions for this must be indicated.  In the guidelines, the return of genetic information is considered to be a “principle.” However, in many research projects it has become customary to not do so based on the conditions separately written in the guidelines.  However, some genome banks have decided to return results because the results of genetic information analysis may be useful not only for research but also for the health of each participant. For example, informed consent in the Tohoku Medical Megabank Project describes the circulation of genetic information as follows: In deciding on whether to return results, the following four conditions will be carefully considered:   1. The information has accuracy and certainty as information for evaluating a health condition 2. The information shows important facts for everyone’s health 3. There is no risk that the proper implementation of research work will be seriously hindered by distributing the information. 4. If there is a significant impact on life and health, then there is an effective treatment. |
| Jordan | c. *The law is silent on return of results; aggregate results are typically returned, but individual results are not returned unless expressly stated in the research protocol.*  In the previous published work, 84.9% were interested to know the results of that research, but with a specific opt-in consent. Similarly, in the unpublished work, 74% respondents wanted to know results when donating biospecimens for biobanking. |
| Mexico | b. *The law is silent on return of results; the expectation is that individual results will be returned unless the participant expressly declines to have the results returned.*  The expectation of receiving results arises from the informed consent provisions. Mexican laws do not intend to govern acts performed in other countries. |
| Netherlands | d. *I am not sure — or other answer*  We do not have such laws as yet, even though scientific journals and media regularly report on research participants in developing countries who contribute to studies but who — as well as others from their country — will probably not get access to the positive outcomes of a study. The *Additional Protocol to the Convention on Human Rights and Biomedicine, concerning Biomedical Research* partially addresses this issue by stipulating that:  (Article 28 — Availability of results) para. 2: “The conclusions of the research shall be made available to participants in reasonable time, on request” and (Article 29 — Research in States not parties to this Protocol): “Sponsors or researchers within the jurisdiction of a Party to this Proto- col that plan to undertake or direct a research project in a State not party to this Protocol shall ensure that, without prejudice to the provisions applicable in that State, the research project complies with the principles on which the provisions of this Protocol are based. Where necessary, the Party shall take appropriate measures to that end.”  The Netherlands is, however, not a party to the Convention and its Protocols. The *Medical Research Involving Human Subjects Act* explicitly prohibits financially compensating research participants to such an extent that this would influence their decision to participate in the study or not (Article 3 under f ). |
| Nigeria | b. *The law is silent on return of results; the expectation is that individual results will be returned unless the participant expressly declines to have the results returned.* |
| Peru | a. *The law requires the return of individual results unless the participant expressly declines to have results returned.*  With the caveats regarding the limited scope of the Peruvian regulatory framework and uncertainties regarding how DTP is governed in the country as stated above, there are requirements for return of individual and aggregate results as well as for community benefit sharing. |
| Poland | c. *The law is silent on return of results; aggregate results are typically returned, but individual results are not returned unless expressly stated in the research protocol.* |
| Qatar | a. *The law [policy] requires the return of individual results unless the participant expressly declines to have results returned.* |
| Singapore | d. *I am not sure — or other answer*  Under the HBRA and the ethical guidelines of the BAC, researchers need to indicate whether or to what extent results will be returned to research participants. |
| South Africa | a. *The law requires the return of individual results unless the participant expressly declines to have results returned.*  DoH guidelines and SAMRC guidelines both require return of results. However, there are concerns regarding lack of regulation to ensure the return of research results to benefit the community that provided the samples and data. |
| South Korea | d. *I am not sure — or other answer*  The Bioethics and Biosafety Act requires that the informed consent document describe the availability period of research results and the details on the access to the information, as explained in Answer 2. This provides the ground for individual participants’ access to the results, while return of results is not mandatory.  The expectation is probably that the individual results will be returned, since past genomic projects returned individual results, which I believe was one of the incentives to participating in genomic research to begin with. For example, the Ulsan 10,000 Genome Project, which launched in 2015, returns individual reports that include genetic variations, ethnicity analysis, haplotype analysis, the “biological age” based on telomere length, and genotypes related to diseases or physical traits. |
| Spain | a. *The law requires the return of individual results unless the participant expressly declines to have results returned.*  Law 14/2007: **49.1.** The subject shall be informed of the genetic data of a personal nature obtained from the genetic analysis according to the terms in which he expressed his will, without prejudice to the right of access recognized in the legislation on the protection of personal data, which may entail the revocation of the prior manifestation of the free will granted. **59. 1.** Without prejudice to the provisions of legislation on the protection of personal data, and in particular Arti- cle 45 of this Law, before issuing con- sent for the use of a biological sample for biomedical research purposes that will not be subject to a process of anonymization, the source subject shall receive the following information in writing: (...) g) The right to know the genetic data obtained from the analysis of donated samples. (...) i) Warning about the possibility of obtaining information relating to his/her health derived from the genetic analyses carried out on the biological sample, as well as about the right to take a position in relation to the communication. |
| Sweden | d. *I am not sure — or other answer* |
| Switzerland | a. *The law requires the return of individual results unless the participant expressly declines to have results returned.*  Article 8 HRA states that “The persons concerned are entitled to be informed of results relating to their health. The information is to be communicated in an appropriate manner. The persons concerned may choose to forgo such information.” This right has a primary goal of helping the concerned person to take a decision on preventive measures and on treatment. If, as part of a research project, a clear result reveals the presence of a disease, the person participating in the project must be kept informed, unless he/she has given up his/her right of being informed. However, it matters in all cases that this result is reliable. As a general rule, the more the disease (existing or likely to develop) is serious and the results are reliable, the more important it is to inform the person. |
| Taiwan | d. *I am not sure — or other answer*  The issues relating to “incidental findings” have been discussed in Taiwan since the World Medical Association adopted and revised the WMA Declaration of Taipei on Ethical Considerations Regarding Health Databases and Biobanks. However, laws, regulations, or practices in Taiwan currently do not directly address these issues. Although the HSRA requires the principal investigator to provide the research protocol, including attribution of research results and uses thereof, said research results and uses are more related to the return of aggregate research results than individual results. |
| Uganda | b. *The law is silent on return of results; the expectation is that individual results will be returned unless the participant expressly declines to have the results returned.* |
| United Kingdom | d. *I am not sure — or other answer* |
| United States | d. *I am not sure — or other answer*  There is currently an active, ongoing debate about the extent to which individual research results should be returned to participants and uncertainty regarding the effect of existing laws on such disclosures (e.g., whether they are prohibited by CLIA or required under HIPAA). Return of results may fall into several categories: public release of study data, return of general (aggregate) study results to subjects, return of individual results to subjects, and return of incidental/ secondary findings to subjects. The extent to which results are returned varies depending on the study. Historically, the return of results to participants was generally limited, but there is currently a trend toward the increased return of individual results. For example, the NIH All of Us Research Program in the U.S. and the U.K. 100,000 Genomes Project will provide individuals access to their research data. This is in line with recent studies showing that participants are generally in favor of receiving individual results. Scholars and researchers opposed to a broader return of individual results often cite increased costs and logistical issues, concerns about validity of the results and potential liability, and a lack of clear protocols for doing so.  Currently, individual or incidental/ secondary research results are generally returned to individuals via one of three mechanisms:  The first is to perform research analyses in laboratories that comply with the C*linical Laboratory Improvement Amendments* of 1988 (CLIA)— a federal statute that aims to ensure the safety and analytic quality of laboratory tests conducted for health care purposes—so that research results can be freely used in clinical care. A second pathway,  for results from non-CLIA research laboratories, is for researchers to confirm results that raise clinical concerns in a CLIA laboratory before return. A third option for results from non-CLIA research laboratories is a clinical hand-off: return research results while advising the participant that clinical confirmation and follow-up are needed before clinical use. In this option, researchers maintain the line between research and clinical care by making a referral for clinical workup rather than venturing a diagnosis based on potentially uncertain research results.  *Law*  CLINICAL LABORATORY IMPROVEMENT AMENDMENTS OF 1988 (CLIA) CLIA prohibits non-CLIA certified laboratories from providing results “for the diagnosis, prevention, or treatment of any disease or impairment of, or the assessment of the health of, human beings.” The Centers for Medicare and Medic- aid Services (CMS) and others have taken the position that “reporting an individual’s research results for any reason is doing so for clinical use and thus needs CLIA certification,” an interpretation that would preclude the return of results from many research laboratories (the majority of which are not CLIA certified). Other scholars disagree, arguing instead that “[w]hen the purpose for return of results is to recommend that the participant seek clinical confirmation and evaluation, rather than for direct use in clinical care, CLIA does not apply [...] Nor does it apply when the goal of returning results is to respect the many nonclinical reasons why participants want their results and data.” These scholars question the authority of CMS to apply such restrictions in the absence of a clear congressional mandate.  THE HEALTH INSURANCE PORTABILITY AND ACCOUNTABILITY ACT (HIPAA) Scholars also disagree about the extent to which HIPAA gives patients a right of access to individual research results. Some argue that “[u]nder the [...] Privacy Rule, research partici- pants have a legally protected right of access to their data and results in the “designated record set” (DRS) at HIPAA-covered clinical and research laboratories,” even if those labora- tories are not CLIA certified. Further, since GINA designates “genetic information” as “health information” under HIPAA, it has been argued that “Congress made clear that these HIPAA access rights include genetic information.”  REVISED COMMON RULE  Although the Revised Common Rule does not directly address the question of whether individual research results should be returned to participants, recent revisions regarding additional elements of informed consent, including broad consent, require researchers to disclose to participants whether results will be returned.  The Revised Common Rule also addresses return of results in the context of a new exemption for secondary research using identifiable biospecimens (or identifiable private information) for which broad consent was initially obtained. The Rule states that this exemption is only available if “[t]he investigator does not include returning individual research results to subjects as part of the study plan.” However, the “provision does not pre- vent an investigator from abiding by any legal requirements to return individual research results.”    *Guidelines*  Several organizations have issued guidelines pertaining to return of individual research results:  AMERICAN COLLEGE OF MEDICAL GENETICS AND GENOMICS (ACMG)  The American College of Medical Genetics and Genomics (ACMG) has published a minimum list of 59 genes that should be screened for in clinical exome and genome sequencing and returned to participants, with their consent, due to their high penetrance and medical actionability. Although the recommendations are primarily directed at clinicians, they have also been adopted by some researchers.  Other organizations have also released recommendations and guidance regarding return of results, including the National Heart, Lung, and Blood Institute (NHLBI) and the Presidential Commission for the Study of Biomedical Issues.  REPORT BY THE NATIONAL ACADEMIES OF SCIENCES, ENGINEERING, AND MEDICINE A recent report by the National Academies of Sciences, Engineering, and Medicine recommends that the decision about whether to return results (and the extent of return) should be made on a study-by-study basis and be clearly described in the research protocol and informed consent process. The report calls for the use of CLIA certified laboratories for results intended to be utilized for clinical decision making. For results not intended to be utilized for clinical decision making, the report allows for the use of non-CLIA certified laboratories, approved research laboratories under a proposed NIH “quality management system” (yet to be established), or after an extensive case-by-case determination about the quality of the results by an IRB. |
